# Role of Radiosurgery/Stereotactic Radiotherapy in Oligometastatic Disease: Brain Oligometastases

**DOI:** 10.3389/fonc.2019.00206

**Published:** 2019-04-04

**Authors:** Rosario Mazzola, Stefanie Corradini, Fabiana Gregucci, Vanessa Figlia, Alba Fiorentino, Filippo Alongi

**Affiliations:** ^1^Radiation Oncology Department, IRCCS Sacro Cuore Don Calabria Hospital, Negrar, Italy; ^2^Radiation Oncology Department, LMU Munich University Hospital, Munich, Germany; ^3^Radiation Oncology Department, General Regional Hospital “F. Miulli”, Acquaviva delle Fonti, Italy; ^4^Radiation Oncology Department, University of Brescia, Brescia, Italy

**Keywords:** oligometastases, radiosurgery, stereotactic radiotherapy, brain metastases, radiotherapy

## Abstract

During the natural history of oncologic diseases, approximately 20–40% of patients affected by cancer will develop brain metastases. Non-small lung cancer, breast cancer, and melanoma are the primaries that are most likely to metastasize into the brain. To date, the role of Radiosurgery/Stereotactic Radiotherapy (SRS/SRT) without Whole brain irradiation (WBRT) is a well-recognized treatment option for patients with limited intracranial disease (1–4 BMs) and a life-expectancy of more than 3–6 months. In the current review, we focused on randomized studies that evaluate the potential benefit of radiosurgery/stereotactic radiotherapy for brain oligometastases. To date, no difference in overall survival has been observed between SRS/SRT alone compared to WBRT plus SRS. Notably, SRS alone achieved higher local control rates compared to WBRT. A possible strength of SRS adoption is the potential decreased neurocognitive impairment.

## Introduction

During the natural history of oncologic diseases, approximately 20–40% of patients affected by cancer will develop brain metastases (BMs) (1). Non-small lung cancer, breast cancer and melanoma are the primaries that are most likely to metastasize into the brain (2, 3).

Recently, the concept of oligometastatic disease was introduced to define a metastatic disease with a low tumor burden, usually represented by 1–5 metastatic sites. Patients with a limited number of brain metastases and controlled extracranial disease are frequently observed in daily clinical practice. In this last subgroup of patients, local-ablative therapies in combination with new molecular agents aim to achieve a longer overall survival (OS) compared to whole brain palliative irradiation (4, 5).

### Biological Aspects of Brain Metastases

Regarding the pathogenesis of metastasis, the oncologic community has generated several hypotheses. A commonly accepted hypothesis is the “seed and soil” hypothesis of Paget, first invoked in 1889 (6). He suggested that the successful growth of metastases and the specific metastatic site preference of certain types of tumors depends on the interactions and properties of cancer cells (the “seeds”) and their specific affinity for the milieu of potential target organs (the “soil”). Metastases result only if the seed and soil are compatible. To date, several studies have confirmed the contemporary relevance of this historic hypothesis. In order to understand the theory, we are briefly going to highlight the process of cancer metastasis, which is sequential and highly selective. First, tumor cells at the primary tumor site must proliferate and initiate angiogenesis. Secondly, tumor cells must invade host stroma and gain entrance into the lymphatics or blood stream to circulate and reach distant organs. Finally, circulating tumor cells must survive the journey through the blood stream and immune and non-immune defenses of the host to extravasate through the microvasculature of target organs (“niche”) to deposit, survive, and grow in a foreign tissue environment (7, 8).

We have to keep in mind that the primary tumors are biologically highly heterogeneous and that metastases can derive from different clonal subpopulations of the primary tumor. For cancer metastasis, cancer stem cells must pass through all stages of the above-mentioned process, including proliferative, angiogenic, invasive, and metastatic steps. Only few cancer cells survive this series of sequential, interrelated steps, as it is highly dependent on the interplay of tumor cells with host factors and organ microenvironment. It has been shown, that cancer cells survive traveling in the circulation and the process of arrest in microcirculatory vessels and extravasion with high efficiency, with >80% of cells successfully completing this process in an experimental setting. Nevertheless, cancer metastasis is known to be complex, and once cells have completed extravasation, they appear much less efficient and more variable at completing subsequent steps in the metastatic process to form macroscopic metastases (9).

The blood-brain barrier of the brain has a specific anatomical and molecular constitution to prevent extravasation of circulating cell types into the brain parenchyma. However, since the brain has no classical lymphatics, hematogeneous metastasis is the only way for tumor cells to get access. Metastasizing cancer cells that arrest in brain microvessels are confronted with a highly alien organ microenvironment. The extracellular matrix, resident parenchymal cells and paracrine signaling molecules, such as cytokines and growth factors differ substantially from other sites of the body (10). Recently, whole-exome sequencing of matched brain metastases and primary tumors first proved the branched evolution of metastases, where all metastatic and primary sites shared common ancestral clones which continued to evolve independently (11–13). Moreover, in >50% of cases, clinically relevant and targetable alterations were found in brain metastases, which were not detected in the primary tumor or extracranial metastases (14). This new evidence is of great importance, especially in the present era of individualized and targeted therapies.

Nevertheless, it seems that there are different patterns of metastatic dissemination. An analysis of clear-cell renal cell carcinomas in a prospective multi-center study (TRACERx Renal) provided a comprehensive picture of the genetic principles and the evolutionary patterns of metastasis. The authors observed distinct models of metastatic dissemination. In cases of rapid progression to multiple sites, metastatic competence is acquired within the most recent common ancestor seeded by primary tumors of monoclonal structure. Usually, this leads to rapid local failure, poor response to systemic therapy and early cancer-related death. In contrast, attenuated progression is characterized by high primary tumor heterogeneity, with metastatic competence acquired gradually and limited to certain subpopulations in the primary tumor. This type of cancer metastasis usually results in initial progression to solitary metastasis, also known as oligometastatic disease, and is characterized by increasing metastatic capacity over time, resulting in more efficient and widespread metastases. This fact underlines the need for aggressive cytoreductive local therapies, in order to minimize the risk of future metastatic seeding from evolving tumors, harboring clones of variable metastatic potential (11, 15, 16).

### Prognostic Factors of Brain Metastases

Several prognostic scores for BMs patients were designed to guide the clinicians' decision-making strategy. In clinical practice, the recursive partitioning analysis classes (RPA), the graded prognostic assessment index (GPA) and the Diagnosis-Specific GPA (DS-GPA) scores (17–21) are routinely used. Gaspar et al. (17) recommended the prognostic index scoring model RPA, which has been developed after evaluating 1,200 patients affected by BMs. Patients were stratified into 3 classes: (i) class I included patients aged up to an age of 65 years with a Karnofsky Performance Status (KPS) >70 and a controlled primary tumor without extracranial metastasis; (ii) class III included patients with a KPS score <70; and (iii) class II included all other cases. RPA classes were associated with different median OS rates: 7.1, 4.2, and 2.3 months for class I, II, and III, respectively (14, 22–24). Recently, Sperduto et al. proposed another prognostic index (GPA), which takes into account 4 clinical criteria (age, KPS, number of BMs, and presence/absence of extracranial metastases) based on data from 5 randomized RTOG trials, including a total of 1,960 patients. A higher GPA score correlated to a better prognosis with a median OS of 11 months, while for GPA scores of 0–1, the OS was 2.6 months (19). Based on an additional analysis, a specific prognostic tool, taking into account the primary histology, was developed (25). The DS-GPA score was correlated to clinical outcome, after stratification by means of diagnosis and treatment. The trial emphasized the heterogeneity in terms of patients' selection, but the usefulness of DS-GPA in clinical practice remains undisputed (20).

Starting from this background, a narrative review of literature was performed evaluating the role of radiosurgery/stereotactic radiotherapy (SRS/SRT) in the treatment of brain oligometastases.

## Methods

We searched PubMed, EMBASE, and Cochrane library for articles published in English language between 1 January 1990 and 1 January 2019. Only randomized studies concerning the irradiation of brain oligometastases were selected.

Inclusion criteria were: randomized studies comparing whole brain radiotherapy (WBRT) vs. SRS/SRT, WBRT vs. WBRT plus SRS/SRT, clinical trials exploring the role of SRS/SRT for 1–5 brain metastases. Exclusion criteria were: articles with no detailed information regarding clinical outcomes, review articles, editorials, articles not written in English language.

## Clinical Data

To assess the role of SRS, several randomized trials have been published in the last decades. In the randomized trial by Kondziolka et al. (26), 27 patients with 2–4 BMs were enrolled and received WBRT alone vs. WBRT and an additional SRS boost. The size of BMs was 2.5 cm or less. WBRT was given up to a total dose of 30 Gy in 12 fractions and the SRS dose was 1 × 16 Gy. Local control rates in patients receiving WBRT alone were 0%, compared to 92% in those receiving a SRS boost, suggesting high local failure with WBRT alone. Median time to local failure was 6 months with WBRT alone compared to 36 months with WBRT and SRS (*p* = 0.0005). In this study, the neurocognitive function was not assessed. In the WBRT plus SRS boost arm, the OS was 11 months, while in the WBRT alone arm the OS was 7.5 months. The Radiation Therapy Oncology Group (RTOG) conducted a similar study (27) from 1995 to 2008. In this trial, 333 patients with 1–3 BMs were randomized to receive WBRT vs. WBRT and SRS. Overall, there was no significant difference in OS between two groups, but a statistically significant advantage for patients with a single lesion. For these cases, the OS increased from 4.9 to 6.5 months with the addition of SRS (*p* = 0.039). It was also observed that in RPA class I patients, survival improved from 9.6 to 11.6 months with the addition of SRS (*p* = 0.045). The results of a follow-up analysis of the RTOG95-08 study were recently published (28). In this study, the RTOG95-08 patients were retrospectively evaluated according to the GPA score (29). The analysis confirmed that there was no OS benefit for patients with 1 to 3 BMs; however, there was a benefit for a subset of patients with a GPA score of 3.5–4.0 (median survival time for WBRT+SRS vs. WBRT alone was 21.0 vs. 10.3 months, *p* = 0.05) regardless of the number of metastases. This result strengthens the observations that SRS, when delivered with WBRT, improves LC and OS in patients with optimal prognostic factors and controlled primary tumors.

At the same time, with the arising of these results, the idea of omitting upfront WBRT in the scenario of oligometastatic BM in favor of SRS/SRT alone evolved, in order to reduce the risk of the neurocognitive deterioration. In this setting, 4 phase III randomized trials (29–32) evaluated the use of SRS alone compared to SRS plus WBRT in patients with 1–4 BMs.

In the Japanese Radiation Oncology Study Group (JROSG 99-1) (30) trial, 132 patients were randomized from 1999 to 2003 to receive SRS with WBRT vs. SRS alone. The inclusion criteria were 1–4 BMs, each with a maximum diameter of 3 cm and a KPS score of ≥70. The primary endpoint was intracranial recurrence rate the secondary points were OS, preservation of neurocognitive function and radiation toxicity. At 12 months follow-up, intracranial recurrence was 76% without WBRT compared to 47% with additional WBRT (*p* < 0.001). The 1 year freedom from new BMs was also improved for the group of patients treated with WBRT (64%) as compared to patients receiving SRS alone (41.5%; *p* = 0.003). Overall, more salvage treatments were required in patients treated with SRS. There were no significant differences in OS, radiation-associated toxicity or death from neurological causes.

Regarding the neurocognitive impact of radiotherapy, a phase III study from the MD Anderson (31) treated patients with 1–3 brain metastases comparing SRS plus WBRT vs. SRS alone. Eligibility requirements were: age ≥18 years, RPA class I or II, KPS ≥70 and 1–3 newly diagnosed BMs. The primary endpoint was neurocognitive function. This was measured as a significant deterioration (5-point drop compared with baseline) in Hopkins Verbal Learning Test-Revised (HVLT-R) at 4 months. An early interim analysis showed a statistically significant decline in learning and memory function at 4 months of 96% for the SRS plus WBRT arm, resulting in an early closure of the trial. Overall, in-brain recurrences were more frequent in the group of patients treated with SRS alone. Within 1 year of follow-up, 73% of patients treated with SRS plus WBRT did not develop new BMs as compared to 27% of patients treated with SRS alone (*p* = 0.0003). In contrast to the JRSOG study (30), the median OS was 15.2 months for SRS alone vs. 5.7 months for SRS plus WBRT (*p* = 0.02). Taken together, the authors concluded that SRS alone with close follow-up is the preferred treatment strategy in patients with newly diagnosed BMs, as improved neurocognitive outcomes and potentially improved OS were reported. In 2010, the European Organization for Research and Treatment of Cancer (EORTC) (20) published the results of a phase III trial, which included patients with 1–3 BMs and a WHO performance status [PS] of 0–2 with a stable systemic disease or asymptomatic synchronous primary tumor. The study compared adjuvant WBRT with observation after SRS or surgery. The primary end-point was time to WHO PS deterioration of more than 2 points. Of 359 enrolled patients, 199 underwent SRS, and 160 underwent surgery. The patients were randomized to observation or WBRT. The median time to WHO PS deterioration of more than 2 was 10.0 months in the observation group and 9.5 months in the WBRT arm (*p* = 0.71). OS was not statistically influenced whether patients received upfront WBRT or not. In patients receiving WBRT, radiotherapy did not improve the duration of functional independence, while it reduced the risk of in-brain recurrence. A secondary analysis of the same study (33) targeted on the health-related quality of life (HRQoL) and showed better HRQoL scores for global health at 9 months in the observation arm as compared to WBRT (*p* = 0.0148). Physical function at 8 weeks, cognitive functioning at 12 months, and fatigue at 8 weeks were improved for patients of the observation group. Recently, the results of the North Coast Cancer Treatment Group (NCCTG-Alliance) N0574 phase III study (21) comparing SRS alone vs. SRS+WBRT in patients with 1–3 BMs (<3 cm) were published. Overall, 208 patients were randomized and the primary endpoint was neurocognitive outcome. The cognitive deterioration was defined as a decline of >1 standard deviation from baseline on at least 1 cognitive test at 3 months. Cognitive deterioration was higher after WBRT with SRS (91.7%) as compared to SRS alone (63.5%, *p* < 0.001). In long-term survivors (≥12 months), cognitive deterioration was more frequent in patients receiving the combined treatment of SRS plus WBRT. The 1 year intracranial control rate was 84.6% with SRS+WBRT and 50.5% with SRS alone. Median OS was 10.4 months for SRS alone vs. 7.4 months with addition of WBRT (*p* = 0.92), but the study was not powered for this endpoint. These results are summarized in Table 1.

**Table 1:** Results by randomized controlled trials (RCT) for 1–4 BMs.

**RCT/Patient number**	**Primary tumor site: %**	**Median age (range)**	**Technique dose**	**Number brain metastases: %**		**Local Control (SRS vs. WBRT+SRS)**	**Distant Control (SRS vs. WBRT+SRS)**	**Overall survival (SRS vs. WBRT+SRS)**	**Neurocognitive Quality of life (SRS vs. WBRT+SRS)**	**Radionecrosis (SRS vs. WBRT+SRS)**
Aoyama (30) SRS (*N* = 67) vs. WBRT + SRS (*N* = 65)	SRS Breast: 4 Lung: 67 Colorectal: 9 Other: 20	SRS: 62.1 y (33–86) WBRT+SRS: 62.5 y (36–78)	Technique: NA SRS alone: 22–25 Gy or 18–20 Gy in 1 fx WBRT: 30 Gy in 10 fx + SRS dose reduced 30%	SRS	WBRT+SRS	72.5% vs. 88.7% 1 y (*P* = 0.002)	36.3% vs. 58.5% 1 y (*P* = 0.003)	28.4% vs. 38.5% 1 y (*P* = 0.42)	MMSE same in both arms	Grade 4: SRS alone 1 cases and SRS+WBRT 2 cases
	WBRT+SRS Breast: 9 Lung: 66 Colorectal: 8 Other:17			1: 49 2–4: 51	1: 48 2–4: 52					
Chang (31) SRS (*N* = 30) vs. WBRT+SRS (*N* = 28)	SRS Breast: 13 Lung: 53 Melanoma: 13 Other: 21	SRS: 63 y (35–82) WBRT+SRS: 64 y (40–78)	PRIMART linear accelerator SRS: 20–24 Gy or 18 or 15 Gy in 1 fx WBRT: 30 Gy in 12 fx	SRS	WBRT+SRS	67% vs. 100% 1 y (*P* = 0.012)	45% vs. 73% 1 y (P = .02)	63% vs. 21%1 y (P = .003)	Statistically significant decline in learning and memory function in WBRT arm	2 cases of grade 4 in SRS alone arm
	WBRT+SRS Breast: 14 Lung: 57 Melanoma: 11 Other: 18			1: 60 2: 23 3: 17	1: 54 2: 28 3: 18					
Kocher (32) SRS (*N* = 90) vs. WBRT+SRS (*N* = 95)	Observation^*^ Breast: 11 Lung: 52 Colorectal: 9 Other: 28	Observation^*^ 61 y (37–80) WBRT^*^ 60 y (26–81)	Linear accelerator and Gamma-knife SRS: 20–25 Gy in 1 fx WBRT: 30 Gy in 10 fx	SRS	WBRT+SRS	69% vs. 81% 2 y (*P* = 0.04)	52% vs. 67% 2 y (*P* = 0.023)	Median OS^*^: 10.9 months vs. 10.7 months (*P* = 0.89)	Decreased HRQoL at 9 months with WBRT arm	SRS alone: 8% SRS and WBRT: 13%
	WBRT^*^ Breast: 12 Lung: 54 Colorectal: 8 Other: 26			1: 68 2: 22 3:10	1: 66 2: 24 3: 10					
Brown (38) SRS (*N* = 111) vs. WBRT+SRS (*N* = 102)	SRS Breast: 9.9 Lung: 72.1 Colorectal: 6.3 Other: 11.7	SRS: 59.8 ± 10.4 y WBRT+SRS: 61.4 ± 10.6 y	Linear accelerator and Gamma-knife SRS alone: 24–20 Gy in 1 fx WBRT: 30 Gy in 12 fx + SRS 22–18 Gy in 1 fx	SRS	WBRT+SRS	72.8% vs. 90.1% 1 y (*P* = 0.003)	69.9% vs. 92.3% 1 y (*P* < 0.001)	Median OS: 10.7 months vs. 7.5 months (*P* = 0.92)	More decline in immediate recall, delayed recall, and verbal fluency in WBRT arm	NA
	WBRT+SRS Breast: 6.9 Lung: 65.3 Colorectal: 4.0 Other: 23.8			1: 49.5 2: 35.1 3: 15.3	1: 54.9 2: 35.3 3:9.8					

* *Including surgical patients. SRS, radiosurgery; WBRT, whole brain radiotherapy; y, year/years; fx, fraction; MMSE, mini-mental state examination; HRQoL, health-related quality of life; NA, not available*.

## Discussion

SRS/SRT without WBRT is an evolving paradigm in the management of patients with limited intracranial disease (1–4 metastases) (34). Historically, the definition of SRS was introduced by Leksell in the 1950s (35), as “a single high-dose irradiation per fraction, stereotactically directed to an intracranial region of interest” to treat BM in a non-invasive way. SRS/SRT procedures have certain characteristics: a well-defined target delineation by means of magnetic resonance imaging, a highly conformal target dose distribution, a steep dose gradient, accurate patient setup and delivery of a high dose of irradiation per fraction. The objectives of these SRS/SRT characteristics are mainly represented by the possibility to decrease the radiotherapy-related intracranial toxicity (through avoidance of WBRT) and to improve tumor control (36, 37).

Concerning the first clinical aspect, Brown et al. published the results of a phase III trial in which patients with 1–3 BMs were randomized to receive SRS or SRS plus WBRT (38). The authors showed that SRS alone resulted in less cognitive impairment compared to SRS plus WBRT. On the other hand, Yamamoto et al. analyzed the role of SRS using Gamma-Knife in 1–10 BMs patients, suggesting that SRS without WBRT in patients with five to ten BMs is non-inferior to the outcome in patients with two to four BMs (39).

The role of WBRT in the management of BMs was recently discussed in two other settings: (i) in the case of resected BMs and (ii) in the BMs from Non-Small Cell Lung Cancer unsuitable for resection or SRS/SRT. In the first clinical scenario, the randomized phase III- NCCTG N107C/CEC3 trial showed that patients who underwent SRS of the surgical cavity had less adverse events and neurocognitive decline compared patients treated with WBRT, without any differences in OS (40). On the other hand, the randomized phase III QUARTZ comparing dexamethasone plus WBRT or dexamethasone alone in case of multiple BMs from Non-Small Cell Lung Cancer unsuitable for resection or SRS/SRT, showed no difference in OS between the two groups (41).

Several other trials tested the impact of SRS/SFRT in case of multiple BMs, reporting no correlation between the number of BMs and OS (42, 43). Thus, the possibility to propose SRS in the setting of BMs is expanding over the numerical well-defined limits of “oligometastases.” As confirmed in the recent NCCN guidelines, SRS could be indicated irrespectively to the number of BMs (not specifically specified) while other aspects, including a good performance status, the overall tumor volume and/or the presence of radioresistant histology are elements which need to be taken into account (44).

In the current review, we focused our search items looking at randomized studies evaluating the potential benefit of SRS/SFRT for brain oligometastases. To date, no difference is observed in terms of OS between SRS/SRT alone compared to WBRT plus SRS. Notably, SRS alone achieves higher rates of LC compared to WBRT. A possible strength of SRS adoption is the potential decreased neurocognitive impairment. In fact, the risk of neurocognitive decline seems to be negligible with SRS alone compared to WBRT, although hippocampal avoidance during WBRT represents a possible technical solution to improve the tolerability of WBRT (45). The upfront SRS approach does not preclude the possibility of performing salvage treatment for new BMs using WBRT or another SRS course. Notably, the upfront omission of WBRT increases the rate of intracranial relapse, in terms of out-of-field appearance of new BMs.

Obviously, this last failure could be related to several factors: (i) the different aggressive biological behavior and genetic heterogeneity of the tumors, (ii) the selective resistance to anti-tumoral drugs, (iii) the poor or non-penetration of drugs across the blood-brain barrier. New systemic therapies are showing promising CNS activity. For this reason, in case of brain oligometastases, the new systemic therapies could act as a “whole brain irradiation” surrogate to control for brain micrometastatic disease, while SRS/SRT can control the macroscopic foci.

In a cost-effectiveness analysis of SRS/SRT alone compared to SRS/SRT with upfront WBRT for BMs, it seems that SRS alone was found to be more cost-effective for patients with 1–3 BMs compared to upfront WBRT plus SRS/SRT (46). The emerging interest to treat patients affected by more than four BMs allowed to introduce a new technology of linac-based SRS/SRT for multiple BMs in daily clinical practice. The main intent of this new technology is to reduce the overall treatment time and the costs for the health systems due to the ability of delivering SRS/SRT for multiple BMs within a single session (47).

In conclusion, the role of SRS/SRT for brain metastases seems to be definitively assessed as a crucial part on the management of BMs patients. SRS/SRT has shown to be a safe and effective treatment procedure, able to pursuit a high level of local control.

## Author Contributions

FA and RM provided the largest writing contribution to the manuscript and edited all sections of the manuscript. AF, SC, FG, and VF performed literature review and wrote multiple sections of the manuscript.

### Conflict of Interest Statement

The authors declare that the research was conducted in the absence of any commercial or financial relationships that could be construed as a potential conflict of interest.

## Abbreviations

BMs: brain metastases
OS: overall survival
RPA: recursive partitioning analysis classes
GPA: graded prognostic assessment index
DS-GPA: diagnosis-specific GPA
KPS: karnofsky performance status
WBRT: whole brain radiotherapy
SRS: radiosurgery
SRT: stereotactic radiotherapy.
